# Experiences of Reframing during Self‐Directed Weight Loss and Weight Loss Maintenance: Systematic Review of Qualitative Studies

**DOI:** 10.1111/aphw.12132

**Published:** 2018-06-01

**Authors:** Jamie Hartmann‐Boyce, Rebecca Nourse, Anne‐Marie Boylan, Susan A. Jebb, Paul Aveyard

**Affiliations:** ^1^ University of Oxford UK

**Keywords:** behavior change, cognitive restructuring, qualitative, systematic review, weight loss

## Abstract

**Background:**

Reframing means changing the way that a person thinks or feels about a weight loss attempt or weight loss maintenance to enhance its experience or facilitate its success. Although participants have described this, it has not been explored in the academic literature. Here, we set out to systematically review qualitative studies to examine the ways in which people use and experience reframing in self‐directed weight loss.

**Methods:**

Seven electronic databases were searched to January 2017 for qualitative studies of adults with overweight or obesity attempting to lose weight or maintain weight loss through self‐directed behavior change. Studies must have contained some information pertinent to reframing. Thematic synthesis was used to identify descriptive and analytical themes from the available data.

**Results:**

We included 23 studies, representing 723 participants. No study focused specifically on reframing. Most studies involved people who had tried to lose weight previously. In the most common examples of reframing, participants spoke of construing previous weight management attempts as “dieting”, whereas in current attempts they used reframing to move away from this concept. Participants spoke of finding reframing helpful because it removed the sense of depriving themselves and instead allowed them to construe the food choices as healthful. Likewise, the language of dieting created a sense of temporary effort, while construing this as a way of life allowed continuation of conscious control over energy balance without the feeling of undue effort. In some cases, these changes were bolstered by change in self‐identity.

**Conclusions:**

Some people construe deliberate weight loss as a form of deprivation and cognitively reframe to avoid the negative emotions this creates and to prevent relapse. Reframing the dietary regimen as about healthy eating and a new way of life made weight control seem less burdensome for these participants and they felt able to maintain their efforts.

## Background

The vast majority of adults who try to lose weight do so outside of formal programs, and without professional support (Santos, Sniehotta, Marques, Carraça, & Teixeira, 2017). However, these self‐directed weight loss attempts have been little researched, particularly with regard to the cognitive and behavioral strategies used by people embarking on these attempts. The Oxford Food and Activity Behaviors (OxFAB) taxonomy has recently been developed to study personal weight control strategies (Hartmann‐Boyce, Aveyard, Koshiaris, & Jebb, 2016). Unlike behavior change taxonomies which focus on the techniques delivered by interventionists, the OxFAB taxonomy aims to describe strategies enacted by the individual. This taxonomy was constructed through qualitative analysis of weight loss resources, consulting with experts in the field, and reviewing existing behavior change technique taxonomies and theories. However, when the taxonomy was applied in a systematic review of qualitative studies of self‐directed weight loss attempts, two additional domains were identified which were not covered in the initial taxonomy (Hartmann‐Boyce, Boylan, Jebb, Fletcher, & Aveyard, 2017). The most commonly coded of these was reframing, and the domain of self‐experimentation was also added.

In this review, we focus on reframing. By reframing, we mean that a person consciously changes the way they think about weight control so as to render it more achievable, bearable, or less emotionally challenging. This is closely related to cognitive restructuring, a technique employed in cognitive behavioral therapy (CBT) (Tolin, 2010). In CBT, the aim is to identify thoughts that are maladaptive because they cause hurtful emotional states and/or lead to dysfunctional behaviors. These thoughts are often automatic, representing habits of mind. In cognitive restructuring, the person is guided to identify the automatic thoughts and challenge their veracity. In so doing, they can replace these with more helpful thoughts, leading to improved emotional health and healthier behavioral patterns.

In pursuing a systematic review of self‐directed weight loss strategies (Hartmann‐Boyce et al., 2017), reframing emerged from participant narratives. However, a literature search has been unable to identify any studies pertaining specifically to reframing or cognitive restructuring in the context of self‐directed weight loss. Therefore, in the first study of its kind, we drew on qualitative studies of self‐directed weight loss to examine how people used reframing and any benefits or harms that it provided for them in this context.

## Methods

Methods for searching, screening, and data extraction are described in brief below but have been published in Hartmann‐Boyce et al. (2017) and Hartmann‐Boyce, Fletcher, Jebb, and Aveyard (2014). These searches were updated and this analysis includes only those studies found in the previous and updated search in which content on reframing was identified. Although Hartmann‐Boyce et al. (2017) describe reframing as a new strategy, the data were not analyzed in that report and it simply noted the phenomenon.

### Searching

Seven electronic databases were systematically searched in January 2017 (CINAHL, EMBASE, MEDLINE, PsycINFO, Science Citation Index Expanded, Social Science Citation Index, Conference Proceedings Citation Index – Science) for qualitative studies using terms related to qualitative research methodologies (those proposed by Cochrane (Booth, 2011)) and terms related to obesity, weight loss, diet, exercise, behavior change and self‐care, which were adapted from a recent systematic review of self‐help interventions for weight loss (Hartmann‐Boyce, Jebb, Fletcher, & Aveyard, 2015). MEDLINE search terms are listed in full on PROSPERO (http://www.crd.york.ac.uk/PROSPERO/display_record.asp?ID=CRD42014012862). Reference lists of included studies and relevant systematic reviews were screened for further studies.

### Inclusion Criteria

The SPICE framework (settings, participants, interest, comparison, evaluation) was used to define inclusion criteria (Andrew, 2006). The settings were unrestricted. Participants included adults who were overweight and had attempted or were attempting to lose weight through behavior change. The interest was the use of reframing by participants in self‐directed efforts to lose weight or maintain lost weight. We included only qualitative studies.

### Screening and Data Extraction

We conducted two rounds of searches. In the first, one reviewer screened titles and abstracts for inclusion, with a sample of 10 per cent checked by a second reviewer (agreement rate 100%). Full text was screened by one reviewer. In the second, due to increased time and resource, two reviewers screened titles, abstracts, and full text for inclusion, with discrepancies resolved through discussion.

Data extraction was conducted independently by two reviewers. We extracted descriptive information about the demographic and socioeconomic characteristics of the population studied as well as information on mean weight status, usually body mass index. We also used a version of the Qualitative Assessment and Review Instrument (QARI) form developed by the Joanna Briggs Institute (2014). This includes information about recruitment processes, methods of analysis, and the role of the researcher in data collection and analysis. Relevant material about reframing was extracted verbatim.

### Analysis

Analysis followed the thematic synthesis approach, set forth by Thomas and Harden (2008) and detailed by Major and Savin‐Baden (2010). Thematic synthesis draws on the methods used in thematic analysis of primary sources, extending them to use in systematic reviews, and consists of three analytical steps: identifying and analyzing first‐order themes (through line‐by‐line coding); synthesising second‐order themes (through organising free codes into related areas to construct descriptive themes); and interpretation of third‐order themes (the development of analytical themes). Analysis was led by JHB and RN, with discussion with other members of the study team throughout. A mix of inductive and deductive approaches was used. First‐ and second‐order themes were identified and analyzed using an inductive approach, with an open coding framework used and new themes gathered from the data as they emerged. The interpretation of third‐order themes was aided by analyzing inductively derived themes through the lens of cognitive restructuring.

## Results

### Characteristics of Included Studies

**Figure 1:**
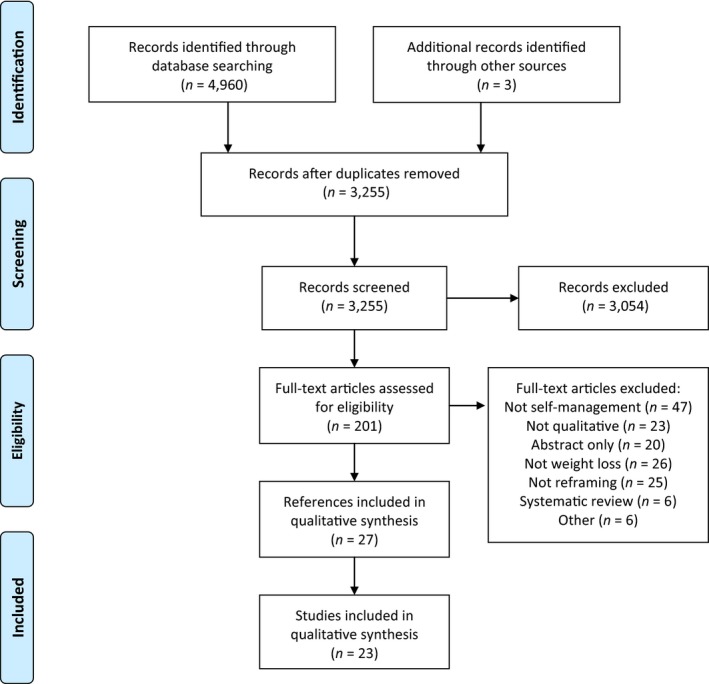
PRISMA diagram of study flow.

Excluding duplicates, searches yielded 3,255 references. Twenty‐three studies met all inclusion criteria, and are included in this analysis, representing 723 participants (Figure 1). Seven of these were unpublished dissertations. Table 1 lists included studies, which are also summarised briefly below.

**Table 1:** Characteristics of Included Studies

Study reference	N	Country	Age	Gender	Weight status	Context	Aims and main findings
Beruchashvili & Gentry, 2006	18	USA	Not described	All women	Not reported	Women who were trying to lose weight or maintain lost weight	Describes factors that aid self‐control—freedom from family responsibilities and social support—and factors that hinder self‐control—seeing food as comfort and its omnipresence.
Couch et al., 2014	47	Australia	Mean 29 years (range 20–43)	All men	BMI 39 (range 30–57)	Men who described their weight loss program in men's health magazine	Men described losing weight by exercise, dietary changes, and associated behavioral strategies.
Davis, 2014	5	USA	All in their 20s	2 men, 3 women	Not reported	US college students actively losing weight	The students separated the processes of weight loss in different stages of the process, felt support was useful to them, their weight loss was adversely affected by stress, and the campus environment.
Frank, 2012	10	USA	Not reported	10% men	Not reported	People who had lost weight and kept it off	Individual growth allowed people to break free of former ways of life and develop a sense of agency over eating.
Granberg, 2006	46	USA	Mean 47 years (range 27–79)	22% men	Mean BMI before weight loss=33, after weight loss=25	People who had sustained weight loss recruited through Weight Watchers or Overeaters Anonymous	The possible future self‐concept and how bodily change reinforced and developed a new self‐concept.
Heading, 2008	19	Australia	Not reported	32% men	21% were healthy weight and the remainder overweight or obese	People who were or had been overweight	Broad ranging: risk logics, embodiment the interplay between the physical, social and subjective body and issues related to adult obesity in remote New South Wales
Hindle & Carpenter, 2011	10	UK	Mean 44 years, standard deviation 12	All women	Mean starting BMI 33	People who had lost weight	Broad ranging: motivation for weight loss and feeling that this was the time, accepting lapses, and lack of motivation to maintain weight because of lack of reinforcement of actions by rewards.
Jaksa, 2011	12	USA	Aged 20 to 57 years	8% men	Not reported	People who had lost at least 20% of body weight and maintained this for at least 2 years	The spectre of the former overweight self‐motivated weight loss maintenance, which was marked by constant vigilance and moderated by trying to find a psychological balance.
James et al., 2016	20	USA	Aged 50 to 70 years	All men	Mean BMI 29, standard deviation 5	Men in general, not necessarily overweight or had lost weight	Description of weight loss strategies used by baby boomer men
Karfopoulou et al., 2013	44	Greece	Mean 32 years, standard deviation 10	41% men	BMI before weight loss was mean 32, standard deviation 7	People who had lost at least 10% of body weight	Contrasts between people who had maintained and regained weight: motivation for weight loss, dealing with eating out, and managing cues to eat.
Klingemann et al., 2016	133	Switzerland	Mean 51 years, standard deviation 14	48% men	Maximum lifetime BMI = 35	People who had lost weight and maintained it versus those who lost and regained weight	The development of behavioral changes in individuals who were able to achieve successful long‐term behavior modification weight loss and maintenance compared to individuals unable to lose substantial weight or to prevent weight regain after weight loss
Macchi, 2007	10	USA	Aged 30 to 45 years	All women	Not reported	Women who used behavioral support contrasted with women who did not	Women approached a “crisis” or threshold, which either prompted action to change themselves or pushed them back to their old behaviors.
McKee et al., 2013	18	UK	Mean age 44 years, standard deviation 13	11% men	Mean current BMI 28	People who had lost 10% of body weight and maintained this contrasted with people who had lost weight and regained it	Contrasts: realistic versus unrealistic goals, the value of routines, self‐monitoring, rules about banned foods, and coping with lapses.
Monaghan, 2008	37	England	Mean age 43 years	All men	Most currently overweight	Interviews explored the value of exercise, not necessarily related to weight control	Men's rejection of the notion of slimness in favor of fitness.
Reyes et al., 2012	29	USA	Mean age 47 years, standard deviation 11	34% men	Mean current BMI 32, standard deviation 5	People who had lost 10% of bodyweight and maintained contrasted with people who regained	Contrasts: maintainers more often continued strategies used during weight loss, weighed themselves regularly, and used productive problem‐solving skills and positive self‐talk.
Ross et al., 2016	20	USA	Mean age 57 years, standard deviation 9	25% men	Mean BMI 27, standard deviation 6	People losing or lost weight through yoga	Yoga helped shift towards healthy eating through mindful eating and stress control, yoga inspired a change in self‐concept, and developed muscle mass.
Stuckey et al., 2011	61	USA	Aged over 21 years	28% men	Not reported	Lost at least 13.6kg and maintained this for a year	People had changed diet by changing food type and quantity, drinking more water, with consistent meal times and planning, regularly used physical activity with monitoring, practised restraint, self‐monitored weight, and deliberately boosted their motivation.
Sweeting et al., 2016	35	Scotland	Mean age 24 years	49% male	Range of BMI from healthy to overweight	All had been overweight in adolescence, some now healthy weight, others still overweight	All were actively controlling their weight with few differences between the healthy weight and overweight participants. Life transitions either helped or hindered weight control.
Thomas et al., 2008	76	Australia	Mean 47 years, range 16 to 72	17% male	Over half had a BMI≥40	Understand experience of people who had a BMI of at least 30 and their approach to life and weight loss	Participants blamed their lack of ability to stick to diets and thought that dieting usually worked
Thorp et al., 2014	11	England	Aged 27 to 59 years	All men	Range of BMIs from healthy to ≥30	To examine men's attitudes towards weight management and behavioral support for weight management	Men set their own standards for what constituted healthy weight for them based on functionality. Men saw weight loss as work and traditional weight management as feminine.
Tyler et al., 1997	80	USA	Mean 34 years, standard deviation 10	All women	Half currently healthy weight, others overweight	Contrasting weight loss methods of African‐American and Euro‐American women	African‐American and Euro‐American women used similar weight loss methods.
Whetstone, 2016	7	USA	Aged 35 to 60 years	All men	All currently had a BMI between 25 and 30	Men who had a BMI>30 and who currently had a BMI<30 maintained for at least a year	To describe understanding of weight and weight loss generally with 14 themes emerging.
Witwer, 2014	12	USA	At least 18 years of age	33% men	Not reported	People who had lost 10% of bodyweight and maintained this for at least a year	To develop a theory of weight loss maintenance, based on transformation of the self.

Overall, study populations were largely homogenous. The mean ages varied between 25 and 60 years, with a mean across all studies that reported this of 43 years. Over half of the studies consisted predominantly of women (mean percentage female 61%). In the studies that reported it, the mean BMI was 31.8 kg/m^2^ (means ranged from 25.8 to 42.5 kg/m^2^). All but five studies were conducted in the USA or UK, with three conducted in Australia, one in Greece and one in Switzerland. Of the studies that reported data on socioeconomic status or ethnicity, populations were predominantly white and of higher socioeconomic status.

Seven studies focused exclusively on weight loss, and eight exclusively on weight loss maintenance. The remainder explored both weight loss and weight loss maintenance. None of the studies explicitly focused on reframing, but through our analysis this relevant theme emerged from verbatim participant quotes. Where data were collected from author summaries or analyses, this is explicitly noted.

The quality of the studies was mixed. The majority employed appropriate data collection. Over half had potential issues with recruitment methods, including recruiting in single settings, thus limiting the range of experiences they collected, or providing insufficient information to judge the appropriateness of the recruitment strategy. Half demonstrated that data analysis was sufficiently rigorous by providing detailed descriptions of the processes used, and in half the role of the researcher had been considered. Understanding the impact of the researcher on data collection and analysis is vital in appraising the quality of qualitative research. In the included studies, this was determined by reflections on the impact of the researchers’ characteristics (e.g. age, gender) on the data collection process, and in one study (Davis, 2014) the author reflected on his own experience of weight loss. Finally, fewer than a third demonstrated that ethical issues had been considered by stating that they had received ethical approval.

### Analytical Themes

The process of thematic coding and synthesis yielded three main analytical themes: reframing eating behaviors; reframing goals; and reframing self‐image.

#### Reframing Eating Behaviors

Eating behaviors was the most common area in which reframing emerged in participant accounts. Accounts of reframing in this context can be grouped into a number of sub‐themes: drawing on past experience; from “diet” to “way of life”; and reframing food.

*Drawing on past experience*: Cognitive restructuring involves examining ways of thinking in order to identify unhelpful beliefs. There were numerous examples of this process in participant accounts of reframing, particularly with regard to eating behaviors. Participants drew on previously negative experiences of losing motivation to continue weight loss and used different language and concepts to facilitate weight management in renewed attempts (Jaksa, 2011; Klingemann, Bucher, Buri, Bolliger‐Salzmann, & Laederach, 2016; Reyes et al., 2012; Sweeting, Smith, Neary, & Wright, 2016). For example, a participant had learned to change the way she thought of food by drawing on past experiences where weight loss efforts had been compromised by thinking of food as celebratory. She explained, “I do kind of stay away from those situations because that's how I started to, when I was first coping, was I took those situations out, and then, um… I didn't want to, ‘Yay, we're going to the most wonderful delicious restaurant’, because I have to think of food differently. So, you know, I don't want to celebrate it” (Jaksa, 2011).

Participants who had experienced weight regain following previous “diets” also drew on this experience to change the terminology they used. A participant from a study of weight loss maintenance explained, “I changed the way I ate. In the past, it was always ‘this is a diet’. And then, when I'm done, I'm going back to the way I ate” (Frank, 2012). Along the same lines, a participant who successfully maintained weight loss after repeated attempts stated that they had tried many “diets” in the past, but were only successful when they decided, “no more diets, develop a healthy eating pattern” (Klingemann et al., 2016). Participants reframed dieting from a fixed‐term effort to a “long‐term venture”, accepting the “permanence” of the behavior changes required (Hindle & Carpenter, 2011; Karfopoulou, Mouliou, Koutras, & Yannakoulia, 2013).

*From* “*diet*” *to* “*way of life*”: Moving away from the word “diet” and towards language such as “the way I eat” or “way of life” was a common use of reframing across studies, and crossed genders, nationalities, and past weight loss trajectories (Couch, Han, Robinson, & Komesaroff, 2014; Frank, 2012; Hindle & Carpenter, 2011; Jaksa, 2011; Karfopoulou et al., 2013; McKee, Ntoumanis, & Smith, 2013; Ross, Brooks, Touchton‐Leonard, & Wallen, 2016; Sweeting et al., 2016; Tyler, Allan, & Alcozer, 1997; Witwer, 2014). “Diet” was often shunned because it evoked either a sense of temporariness, a sense of restriction, or both. In a British study, a now successful weight loss maintainer who had repeatedly lost and regained weight explained, “I went this time with a more rounded attitude of this is going to be a way of life, and this is the way I'm going to live, I'm not just doing this for this diet I'm doing this forever you know. And I think this was why I thought I've got to be more relaxed about it.” A second participant from the same study explained, “I went with the belief that this wasn't a diet, but what I'd got to do was change my way of eating” (Hindle & Carpenter, 2011). In a Greek study of weight loss maintainers following a Mediterranean diet, a participant explained, “… to realise that the X diet plan they will give you has to be a way of life, and not a 6‐month period you're following it and then going back, to how you were before” (Karfopoulou et al., 2013). In another British study of weight loss maintenance, a participant elaborated on this concept by couching diet as a temporary term: “I mean people say they are on a diet when they are a member of a slimming club sometimes but I don't think that's what you're on, you don't ever intend to go back to what you were doing before so that is not a diet you have just changed the way you eat now… I think you've got to tell yourself you're not on a diet you're just changing your way of life … you're not on a diet which is a temporary thing—you've got to educate yourself to eat differently all the time” (McKee et al., 2013).

In the above quote, the shift away from “diet” to “changing the way you eat” is also twice coupled with the word “just”, implying that “changing the way you eat” may connote an easier or simpler process for this participant than “dieting”. This was reflected in other narratives, in which participants elaborated further on why they moved away from diet terminology. In an American study of weight loss maintenance, a participant explained, “It's not a diet. I changed the way I ate. … it's not a diet, and, and I hate that word. Because that very word, um, connotes (*sic*) deprivation. So I've, I try hardly ever to say that word. Um, instead I'll say my food plan, or my food program” (Frank, 2012). In other instances, participants felt that “diet” connoted a strictness or set pattern that was not sustainable. In an Australian study, a 24‐year‐old participant explained, “The word ‘diet’ is toxic. You have to allow yourself the odd piece of cake or slice of pizza on special occasions” (Couch et al., 2014). Finally, in a Scottish study looking at weight control in the transition to adulthood, a female participant explained “… and then I dieted and just started … not so much dieted, it just changed the way I ate, rather than actually following Weight‐Watchers or anything like that” (Sweeting et al., 2016).

*Reframing the meaning of food*: As well as moving away from the word “diet”, participants also spoke of reframing the meaning of food itself. In some cases, participants made the deliberate decision to reframe some food as something with negative connotations, whether as “toxic”—“to see that kind of food as poison to my system, if I eat it, it will hurt me” (Jaksa, 2011), as a “crutch” (Beruchashvili & Gentry, 2006), or as “drugs”—with bread rolls as “white heroin” and unhealthy foods described as “addictive” (Jaksa, 2011). In an American study, a female participant spoke of finding it difficult to reframe the meaning of food as a “vice”; she had previously thought of it as a “comfort item” and found it “hard” that it was no longer available to her (Macchi, 2007). In other instances, food was reframed to be more functional, using the term “fuel”; a participant in an American study of successful weight loss maintenance explained, “I have to think of food differently. So, you know, I don't want to celebrate it. You know? I just think it's a fuel, like I don't celebrate putting gas in my car” (Jaksa, 2011). This is reminiscent of the participant quoted above who deliberately moved away from thinking about food as celebratory (“Drawing on past experiences”). In stark contrast, in a separate study, an American man who was overweight and lost weight through yoga discussed beginning to “think of eating more as dining and less as fueling up” (Ross et al., 2016), in other words moving away from viewing food as functional, and towards viewing eating as more of an occasion. These examples illustrate how different metaphors may work for different people.

#### Reframing Behavioral Goals

As well as reframing the behavioral elements of weight management, reframing was also used to reframe weight loss goals—in particular, to reframe their weight loss attempt as an attempt to improve their health and lifestyle, rather than simply to lose weight (Davis, 2014; Heading, 2008; Klingemann et al., 2016; Monaghan, 2008; Ross et al., 2016; Thomas, Hyde, Karunaratne, Kausman, & Komesaroff, 2008; Thorp, Elliott, & Ellahi, 2014; Whetstone, 2016). In an Australian study, a participant explained, “Let's not focus on weight loss, let's focus on health and lifestyle … it has to be about empowering bigger people to make choices that will benefit their lifestyle, their life. It shouldn't be about weight loss, let's take it away from the kilos and the pounds and you've got to lose, lose, lose” (Thomas et al., 2008). Similarly, in a study of adults with obesity living in rural Australia, a female participant explained, “[I stopped thinking of it as] trying to lose weight and looked after my health instead and that helped me” (Heading, 2008). In a study of obesity discourses amongst British males, Monaghan drew parallels with these views and the Health at Every Size (HAES) movement (see Discussion), explaining how, in the participants he studied, “physical fitness is prioritised over slimness for biomedical health and wellbeing” (Monaghan, 2008).

#### Reframing Identity

Finally, some participants reframed not the weight loss attempt or associated behaviors, but the way they viewed themselves. In an American study of weight loss maintenance, a male participant explained, “I decided that I was going to become a runner. And then when I changed my mindset about that, that I eat healthy because I'm a runner and if I don't eat healthy then I'm not going to run as well, it makes it seem less like something I'm forcing myself to do than something I enjoy doing” (Reyes et al., 2012). In a second American study exploring the role of possible selves in efforts to maintain weight loss, Granberg (2006) explains how participants enacted “a canonical self‐transformation narrative”. These narratives shifted over time, with reframing playing a crucial role in facilitating these shifts. The author explained, “When the outcome failed to fulfill their expectation, this narrative came into doubt. If weight loss was to be sustained and if the identity transformation was to continue, another satisfactory narrative had to be found …. Among these respondents, the major form of this narrative involved a reframing of both weight loss and its disappointments as a mechanism for personal growth.” One participant elaborated, “If anything, losing the weight caused me to become a person who dealt with the problems in my life … It caused me to confront some of the issues that I previously had been hiding from” (Granberg, 2006).

## Discussion

### Summary of Main Findings

In this qualitative review of self‐directed weight loss, participants did not report using reframing because they had been advised to do so but it emerged through deliberately reflecting on their past experience. Evoking cognitive restructuring, participants described consciously identifying unhelpful beliefs in order to overcome barriers they had encountered in previous weight loss attempts. Often, this involved moving away from the language of “dieting” and “weight loss”, but some participants also reframed their own identities in an effort to manage their weight.

### Strengths and Limitations

To our knowledge, this is the first study of any kind to focus specifically on participant accounts of reframing and its value as a weight management technique. A scoping search run in March 2017 uncovered no articles focusing on reframing or cognitive restructuring in weight management, and in our work on the OxFAB taxonomy, reframing as a weight loss technique did not feature in behavioral weight loss programs, or self‐help books, nor was it suggested by experts as a technique to support successful weight control. Consequently, we systematically reviewed the qualitative literature for examples of this technique. The strength of this approach lies in its focus on direct experiential evidence from participants in the form of verbatim quotations. As such, we found evidence for reframing even in studies in which it was not the focus of the research. In some ways this divergent focus was also a limitation of the approach because the quotes were often short and the participants did not elaborate on the topic (as they were not prompted to do so). Moreover, because the focus of the original studies was not on reframing, we have no information about what prompted reframing and how participants achieved this. Finally, a lack of focus on the cognitive framing of a weight loss attempt leaves the counterfactual almost unexplored. We presume that construing a weight loss attempt as a diet and an effortful period of striving could be adaptive for some people, but because this was commonplace it was left unexamined. As such, these data do not imply that reframing should be seen as universally helpful; only that it is a technique that some people settle on and find helpful, but is not generally recognised by the field. However, this review demonstrates clear evidence that reframing weight loss is important for some people and this warrants further investigation.

An additional strength is that we have focused on self‐directed weight loss attempts, which have been under‐researched in comparison with people's experiences of formal weight loss interventions. This may be why reframing has not yet emerged as a prominent theme in qualitative literature on weight loss—previous syntheses focusing on weight loss programs may have been constrained by the aims of process evaluations or by the language used in the programs themselves, whereas our broader focus allowed this theme to emerge and be explored in more depth. However, our focus on self‐directed weight loss can also be viewed as a limitation; reframing may also be used by participants engaged in supported weight loss attempts, and this may be an avenue for future research.

A further limitation of this review is the quality of the included studies, in particular with regard to the consideration of ethical issues, recruitment methods, coding of data, and consideration of the role of the researcher. Given the nature of the available data, in some instances it is difficult to determine whether the content of the studies reflects the experiences of the participants, or if the studies’ results have been tailored based on the interests of the researchers. A minority of the papers reviewed spoke about reframing, and given the above limitation it is difficult to know whether that is due to limited use amongst participants or due to researchers focusing on other aspects of the weight loss process. We coded verbatim participant quotations, increasing our confidence that the findings we synthesise here draw upon the experiences of the participants interviewed. However, this review relied on studies where reframing was not the focus. As such, it is likely that participants who had reframed a weight loss attempt are likely to report doing so and only those who found it helpful to do so are likely to be still using this technique. People who reframed and found it unhelpful or who find the “diet” framing helpful may not remark on this and, without a focus on reframing, these experiences may have been missed. In the same way, while the quotes seemed to hint at the value of reframing for weight loss maintenance, there were no data that really spoke clearly to the distinction between weight loss and weight loss maintenance and this distinction appeared less sharp in participants’ minds than is the case for weight control researchers.

### Findings in Context

Although cognitive restructuring has long been recognised as a behavior change technique within the context of CBT, there is little evidence of reframing or cognitive restructuring being formally recognised in a weight control context. The terms “diet” and “weight loss” are relatively ubiquitous in healthcare dialogue on weight management and in weight loss programs, although sociological and related literature has a more critical discourse on these terms. Moreover, there are very few examples of CBT being integrated into behavioral weight loss programs (Castelnuovo et al., 2017). CBT has been used in the context of coexisting binge eating disorder and obesity and alone it produces little weight loss, but with a behavioral program is more effective (Peat et al., 2017). The Health at Every Size (HAES) Movement has parallels with some of the information included in this review. HAES explicitly focuses on achieving good health without placing an emphasis on weight or food restrictions, tying into themes emerging from this review on shifting discourses from “weight” to “health”. Work from Deborah Lupton on obesity discourse and politics has previously highlighted how HAES positions itself in direct opposition to dominant discourses of obesity science (Lupton, 2012). Although it is interesting to note the similarities between participant accounts of reframing and some of the discourses used within HAES and HAES interventions, the experiences of reframing presented here should not be conflated with HAES, as HAES explicitly moves away from weight loss as a goal. In the accounts in this review, the use of health‐based discourses did not preclude weight loss but instead appeared to facilitate weight loss for some participants by strengthening a long‐term goal of improved health.

Some of Lupton's other work on obesity discourses also resonates with participant accounts presented in this review. For example, Lupton cites examples of focusing on physical fitness and muscular strength instead of weight loss as a way to enable people from the “fat pride” movement and people who identify as feminists to feel as if they had not contravened their principles by trying to lose weight (Lupton, 2012). Lupton highlights this focus on strength as opposed to weight as a feminist issue, while Monaghan discusses the shift in focus from weight loss to physical strength as a uniquely male example of reframing (Monaghan, 2008).

## Conclusions

Existing behavior change technique taxonomies and many weight loss programs do not include reframing. It appears widely overlooked in empirical research on weight loss interventions and in research on self‐directed weight loss attempts. It is clear from this review that we may benefit from understanding more about reframing and in particular the processes people go through when employing this technique, including their motivations for doing so, and where the technique may and may not converge with the processes involved in cognitive restructuring. Obesity is a chronic, relapsing condition and future weight management research and interventions may benefit from considering the ways in which language could be used to break down some barriers people face when embarking on weight loss attempts, especially after previous unsuccessful efforts. This may promote more successful weight control—both initial loss and weight loss maintenance. This does not imply that much of the *content* of the weight management interventions needs to change. However, programs may benefit their users if they aid them to change the language and metaphors about energy balance and reflect on past experiences and emotional reactions to previous weight loss attempts. Ultimately, the true test of the value reframing as an aid to weight loss will come from intervention studies in which participants are helped to reframe their weight loss attempts compared with usual care.

## Competing Interest

The authors declare that they have no competing interests.
